# The adeno-associated virus rh10 vector is an effective gene transfer system for chronic spinal cord injury

**DOI:** 10.1038/s41598-019-46069-z

**Published:** 2019-07-08

**Authors:** Yutaka Hoshino, Kenji Nishide, Narihito Nagoshi, Shinsuke Shibata, Nobuko Moritoki, Kota Kojima, Osahiko Tsuji, Morio Matsumoto, Jun Kohyama, Masaya Nakamura, Hideyuki Okano

**Affiliations:** 1 Department of Orthopedic Surgery, Keio University School of Medicine, 35 Shinanomachi, Shinjuku-ku, Tokyo 160-8582 Japan; 2 Department of Physiology, Keio University School of Medicine, 35 Shinanomachi, Shinjuku-ku, Tokyo 160-8582 Japan; 3 Electron microscope laboratory, Keio University School of Medicine, 35 Shinanomachi, Shinjuku-ku, Tokyo 160-8582 Japan

**Keywords:** Spinal cord injury, Genetic vectors

## Abstract

Treatment options for chronic spinal cord injury (SCI) remain limited due to unfavourable changes in the microenvironment. Gene therapy can overcome these barriers through continuous delivery of therapeutic gene products to the target tissue. In particular, adeno-associated virus (AAV) vectors are potential candidates for use in chronic SCI, considering their safety and stable gene expression *in vivo*. Given that different AAV serotypes display different cellular tropisms, it is extremely important to select an optimal serotype for establishing a gene transfer system during the chronic phase of SCI. Therefore, we generated multiple AAV serotypes expressing *ff*Luc-cp156, a fusion protein of firefly luciferase and Venus, a variant of yellow fluorescent protein with fast and efficient maturation, as a reporter, and we performed intraparenchymal injection in a chronic SCI mouse model. Among the various serotypes tested, AAVrh10 displayed the highest photon count on bioluminescence imaging. Immunohistological analysis revealed that AAVrh10 showed favourable tropism for neurons, astrocytes, and oligodendrocytes. Additionally, with AAVrh10, the area expressing Venus was larger in the injury epicentre and extended to the surrounding tissue. Furthermore, the fluorescence intensity was significantly higher with AAVrh10 than with the other vectors. These results indicate that AAVrh10 may be an appropriate serotype for gene delivery to the chronically injured spinal cord. This promising tool may be applied for research and development related to the treatment of chronic SCI.

## Introduction

Traumatic spinal cord injury (SCI) results in severe functional deficits due to the limited regenerative capacity of the central nervous system (CNS). The annual incidence of SCI is 250,000 to 500,000 cases worldwide^1^. To date, there are few effective treatments for SCI in clinical settings; therefore, experimental interventions have been extensively examined in animal models. Among these interventions, we and others have reported the efficacy of cell transplantation therapy using embryonic stem cell (ESC)-derived or inducible pluripotent stem cell (iPSC)-derived neural stem precursor cells^2–4^ or mesenchymal stem cells^5^. In addition to those procedures, therapeutic intervention has also been reported using chemical compounds and trophic factors, including riluzole^6–8^, glibenclamide^9^, granulocyte-colony stimulating factor (G-CSF)^10,11^, hepatocyte growth factor (HGF)^12,13^, an anti-Nogo antibody^14–16^, chondroitinase ABC (c-ABC)^17–19^, and a semaphorin 3A inhibitor^20^. Despite these beneficial effects, most of these studies focused on the acute to subacute phase of SCI as the preferred timing for therapeutic interventions, and treatment options for the chronic stage remain limited^21^. Several studies performed intrathecal c-ABC administration with combinatory therapy for chronic SCI and reported a slight improvement of motor function^22,23^. However, catheter implantation was technically demanding, and the release of this enzyme was temporary over only one week.

Gene therapy is expected to be a promising treatment because it can directly deliver the therapeutic gene product to the target tissue. Moreover, this procedure is effective after a single injection and is less expensive than conventional drug administration^24^. For SCI, a few studies have reported that gene delivery of neurotrophic factors using adenovirus and lentivirus vectors induced histological improvement and functional recovery in the rat SCI model^25,26^. Although therapeutic interventions using these viral vectors have beneficial effects, there remain risks, including the host immune response^27^ and the theoretical potential for oncogenesis caused by insertional mutation into the host genome^28^. Considering the validity of this approach as not only an experimental tool but also a system applicable to clinical settings, it is important to select a vector that can safely and efficiently transfer the gene of interest to targeted cells or tissues.

AAV vectors have emerged as powerful vehicles for gene delivery. These vectors have been proven to be transferred to both dividing and non-dividing cells with favourable characteristics, including low host genome integration, low immunogenicity and long-lasting gene expression *in vivo*^29^, all of which are significant advantages over other viral vectors. In fact, gene therapy using AAV vectors has been widely utilized for incurable diseases^30–34^. Historically, AAV2 is the serotype that was initially identified, and various AAV serotypes have been generated by cross-packaging of the AAV2 inverted terminal repeat (ITR). Given that different AAV serotypes display different cellular tropisms^35^, it is critical to select an optimal serotype for the target tissues or cells. Regarding the spinal cord, several serotypes, such as AAV1, 5^36^, 6, and 9^37^, were reported to show increased tropism for the intact parenchyma. For SCI, the characteristics of AAV serotypes were examined only during the hyper-acute phase^38,39^, and there are no conclusive reports about the suitable serotypes for the injured spinal cord in the chronic phase. Considering phase-dependent changes in the intraspinal environment^40^, the appropriate serotype of AAV for targeting chronic SCI should be comprehensively examined as a tool for therapeutic intervention. Therefore, the purpose of this study was to identify the most efficient and highly transduced AAV vector for chronic SCI.

## Results

### Establishment of a reporter system for analysing cell type-specific tropism of AAV

**Figure 1:**
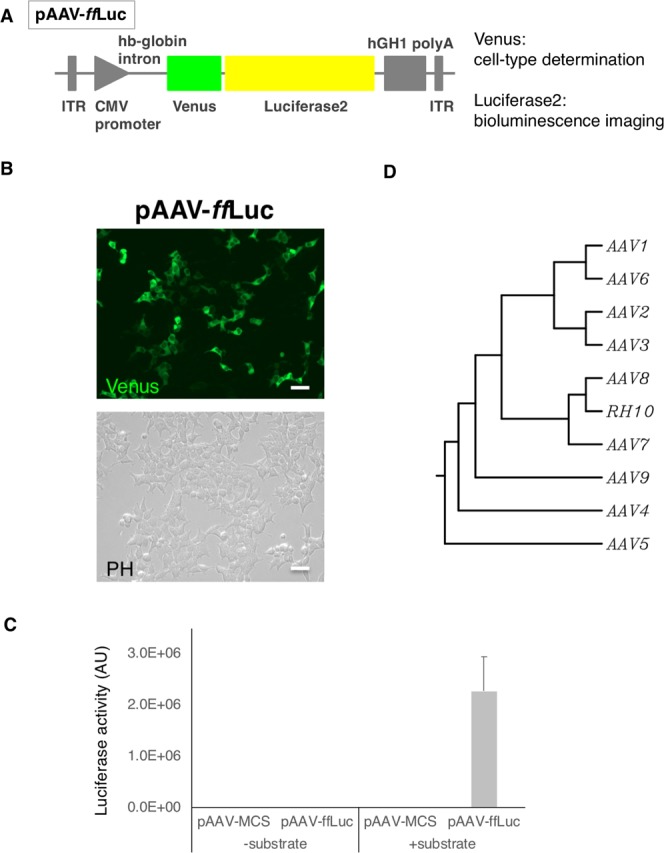
Establishment of a reporter system for analysing the cell type-specific tropism of AAV. (**A**) Diagram of the AAV vector. The Venus-luciferase 2 reporter gene cassette (*ff*Luc-cp156: *ff*Luc) was used and inserted into pAAV-MCS, which contains AAV2 ITRs, the CMV promoter, the human β-globin intron and the human growth hormone polyadenylation signal. (**B**) Representative phase contrast and fluorescence images of HEK293T cells after transient transfection of pAAV-*ff*Luc. Scale bars, 50 μm. (**C**) Luciferase activity of HEK293T cells after transfection of pAAV-*ff*Luc. Luciferase activity was observed only in the cells transfected with pAAV-*ff*Luc. (**D**) Phylogenetic analysis based on the amino acid sequence of the VP1 capsid protein for each serotype.

To analyse the efficacy of transduction mediated by AAV, we generated an AAV vector engineered to express *ff*Luc-cp156, a fusion protein of firefly luciferase and the fluorescent protein Venus^41,42^, as a reporter (Fig. 1A). Since *ff*Luc-cp156 is applicable to bioluminescence systems in addition to its use as a fluorescent protein, we could screen for desired AAV serotypes based on photon yield, as indicated by bioluminescence imaging, and cell-type specificity, as indicated by Venus expression in AAV-transduced tissues. To validate this system, we initially introduced pAAV-CMV-*ff*Luc into HEK293T cells by transient transfection and examined the reporter activity. As shown in Fig. 1B and C, both Venus-driven fluorescence and luciferase activity were detected in cells carrying the reporter. Thereafter, we used the plasmid to generate AAV serotypes for screening for desired serotypes for SCI. In brief, AAV serotypes were analysed based on the amino acid sequence of the VP1 capsid protein and classified into typical clades as described in previous reports^43^ (Fig. 1D). Even with high sequence similarity, some serotypes have been reported to exhibit different tropisms^44^. Therefore, we further characterized each serotype *in vivo*.

### AAV 1-9 and rh10 were successfully transduced in the intact spinal cord in mice

**Figure 2:**
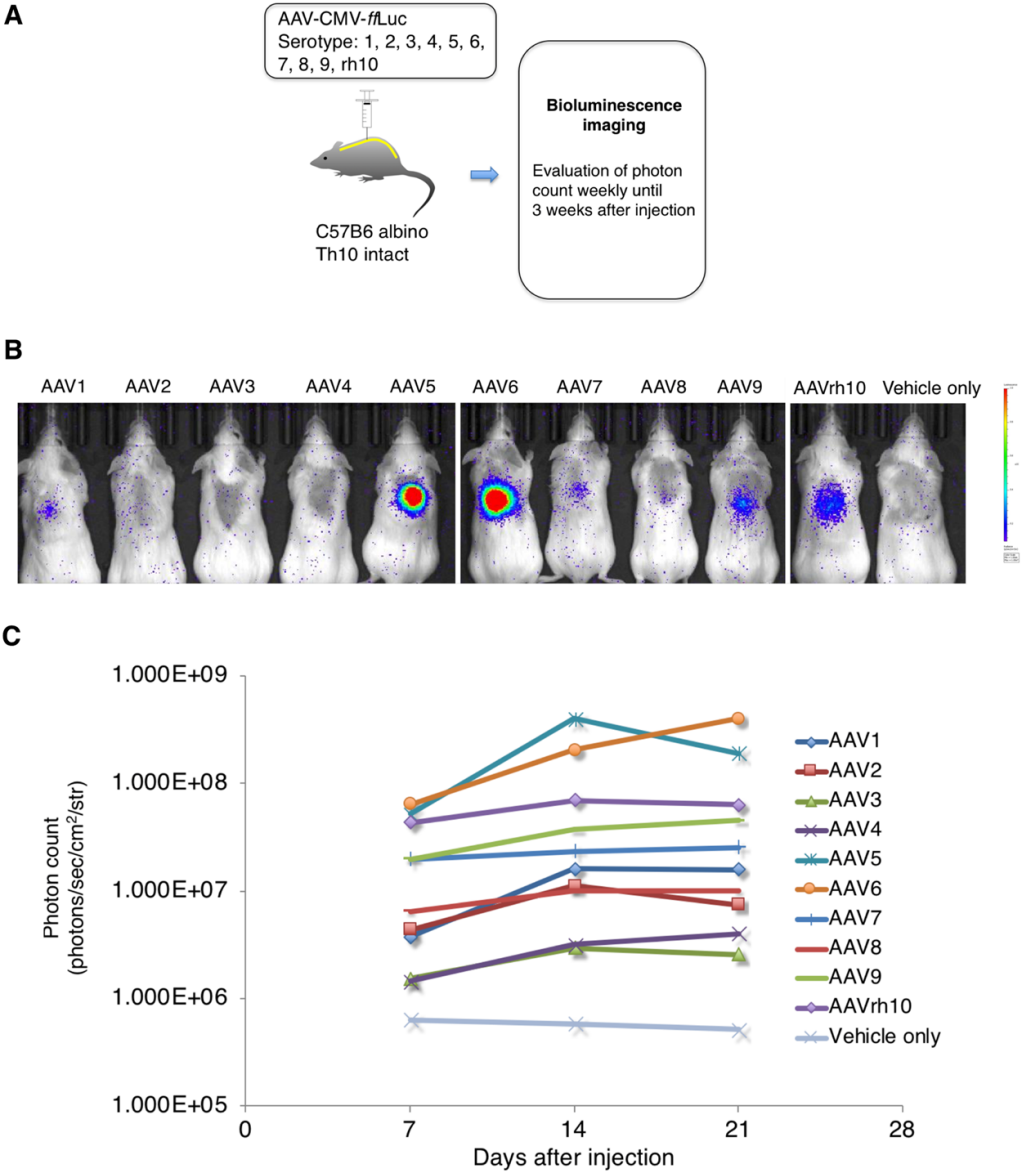
Efficient transduction of the intact mouse spinal cord by AAV1-9 and AAVrh10. (**A**) Schematic diagram of AAV-*ff*Luc injections into the intact spinal cord. AAV-*ff*Luc serotypes 1-9, and rh10 were injected into intact mouse spinal cords to validate and evaluate gene expression *in vivo*. A mouse injected with vehicle only was used as a negative control. (**B**) Representative *in vivo* BLI of a mouse at 3 weeks post injection of AAV-*ff*Luc. (**C**) Photon counts from *in vivo* BLI derived from AAV-*ff*Luc until 3 weeks post injection.

To examine the efficacy of transduction of each AAV serotype *in vivo*, the AAV vectors were injected into the parenchyma of intact mouse spinal cords, followed by bioluminescence imaging (BLI) for three weeks after injection (Fig. 2A). As shown in Fig. 2B, we detected *ff*Luc-cp156-driven chemiluminescence activity in the spinal cord after the injection, while some AAV serotypes displayed almost negligible reporter activity. Among the serotypes, AAV5, 6, 9, and rh10 showed the highest photon counts three weeks after the injection (Fig. 2B and C), which was largely consistent with previous reports of AAV tropism using an intact spinal cord model^36,37^. In a prior study using a model of hyper-acute SCI, AAVrh10 was superior to AAV9 in injured tissue, as indicated by higher neuronal and glial tropism^38^. Based on the results from our study and other studies, we selected AAV5, 6, and rh10 for administration in the injured spinal cord during the chronic phase.

### AAVrh10 effectively transduced cells in the chronically injured spinal cord

**Figure 3:**
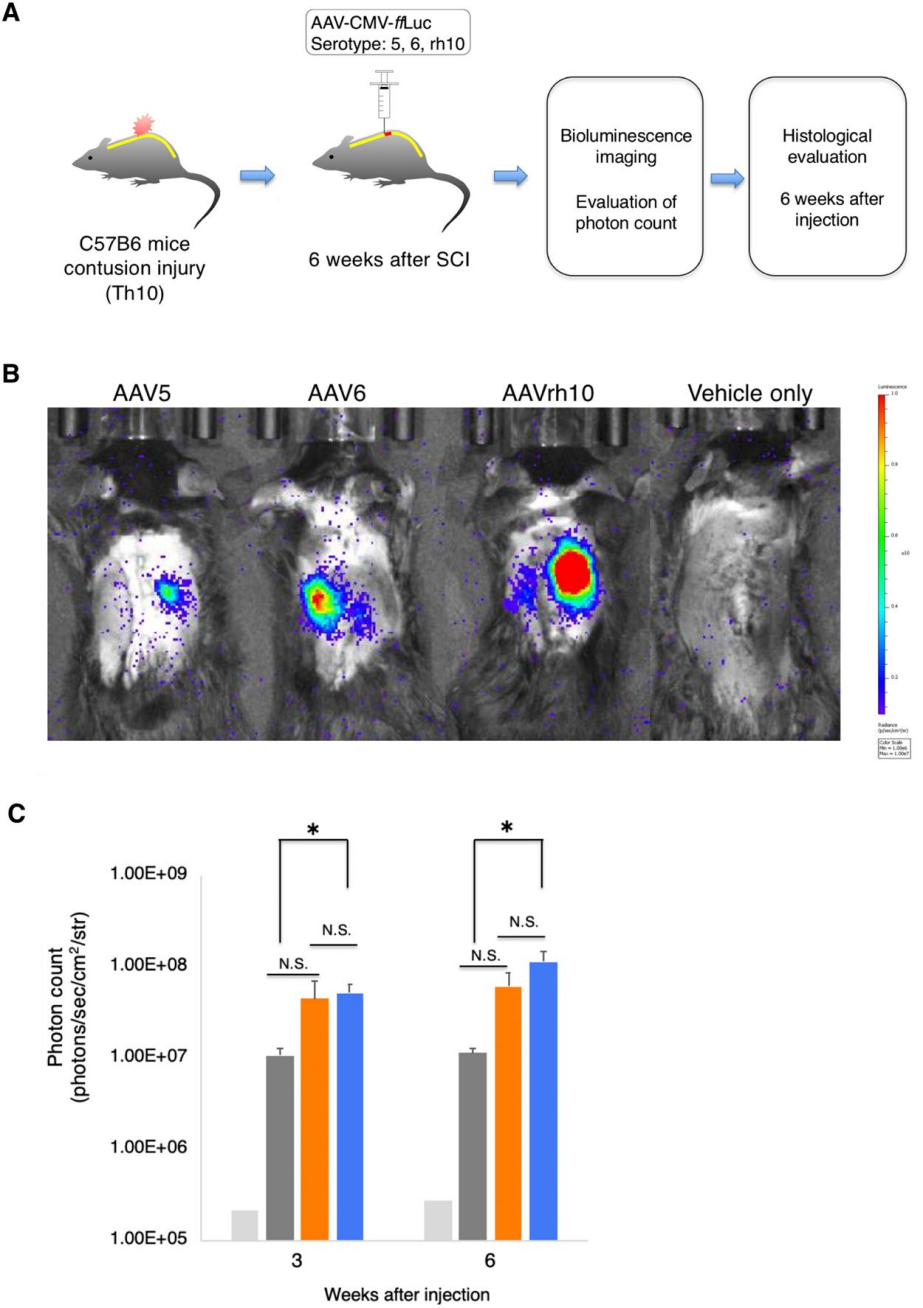
Chronically injured spinal cord was effectively transduced by AAVrh10. (**A**) Schematic diagram of AAV-*ff*Luc injections into the chronically injured mouse spinal cord. AAV serotypes 5, 6, and rh10 were injected into the chronically injured spinal cord. Mice injected with vehicle only were used as negative controls. (**B**) Representative *in vivo* BLI of a mouse at 6 weeks post injection of AAV-*ff*Luc. (**C**) Photon counts from *in vivo* BLI derived from AAV-*ff*Luc until 6 weeks post injection. AAVrh10 exhibited a higher photon count than AAV5 at 6 weeks after injection. *p < 0.05 and not significant (N.S.) according to one-way ANOVA with the Tukey-Kramer test. Data are presented as the mean ± SEM. n = 4 for each serotype.

To evaluate the properties of AAV5, 6, and rh10 in chronic SCI, we generated contusion injuries in the mice at the 10th thoracic level. Six weeks after the injury, intraparenchymal injection of the AAV vectors was performed at two points immediately rostral to the injury epicentre (n = 4, each serotype) (Fig. 3A). According to BLI, AAVrh10 showed the highest photon count among the serotypes. In fact, AAVrh10 exhibited significantly higher luminescence than AAV5 at five and six weeks after the injection (Fig. 3B and C).

**Figure 4:**
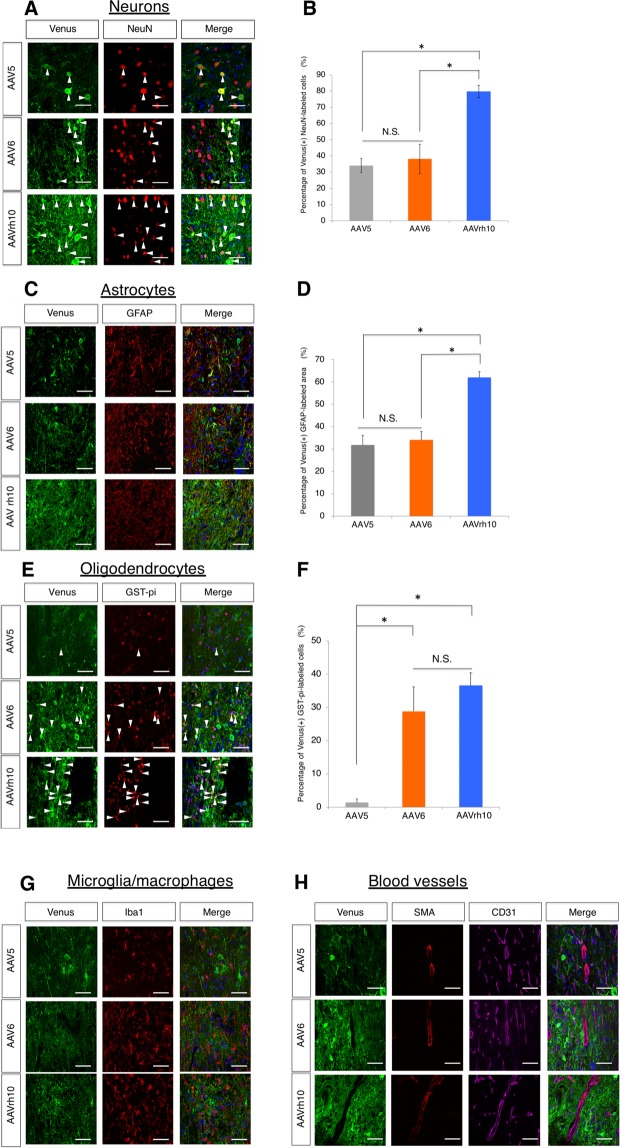
Cellular tropism of AAV5, 6, and rh10 in the injured spinal cord during the chronic phase. Sections were immunostained with an anti-GFP antibody, together with cell type-specific markers for neurons (NeuN) (**A**), astrocytes (GFAP) (**C**), oligodendrocytes (GST-pi) (**E**), microglia/microphages (Iba1) (**G**) or blood vessels (CD31 and SMA) (**H**) at 6 weeks after AAV injection. Scale bars, 50 μm. Quantification of the transduction rates in neurons, astrocytes and oligodendrocytes is shown in (**B**), (**D**) and (**F**), respectively. The green fluorescence of representative images was adjusted. Arrowheads indicate examples of double-positive cells. *p < 0.05 and not significant (N.S.) according to one-way ANOVA with the Tukey-Kramer test. Data are presented as the mean ± SEM. n = 4 for each serotype.

Since differences among serotypes are considered to affect cellular tropism at the cell entry and post-entry levels and result in various transduction characteristics^45,46^, histological analysis was performed to evaluate the cell selectivity of each AAV. Accordingly, cell lineages, including neurons, astrocytes, oligodendrocytes, microglia/macrophages, and blood vessels, were evaluated for Venus expression by using cell type-specific markers. As shown in Fig. 4A–D, AAVrh10 displayed significantly higher tropism than AAV5 and AAV6 for neurons (79.7 ± 3.8% vs. 34.1 ± 4.3% vs. 38.2 ± 9.1%) and astrocytes (62.0 ± 2.7% vs. 31.8 ± 4.3% vs. 34.1 ± 3.8%). For oligodendrocytes, the transduction rate of AAV5 was significantly lower than that of AAV6 or AAVrh10 (1.4 ± 1.1% vs. 28.9 ± 7.2% vs. 36.6 ± 3.9%) (Fig. 4E and F). On the other hand, transduction to microglia/macrophages and blood vessels was hardly detected (Fig. 4G and H). Taken together, these results demonstrate that AAVrh10 is more effective than the other AAV vectors for manipulating gene expression in neurons, oligodendrocytes, and astrocytes in chronic SCI.

**Figure 5:**
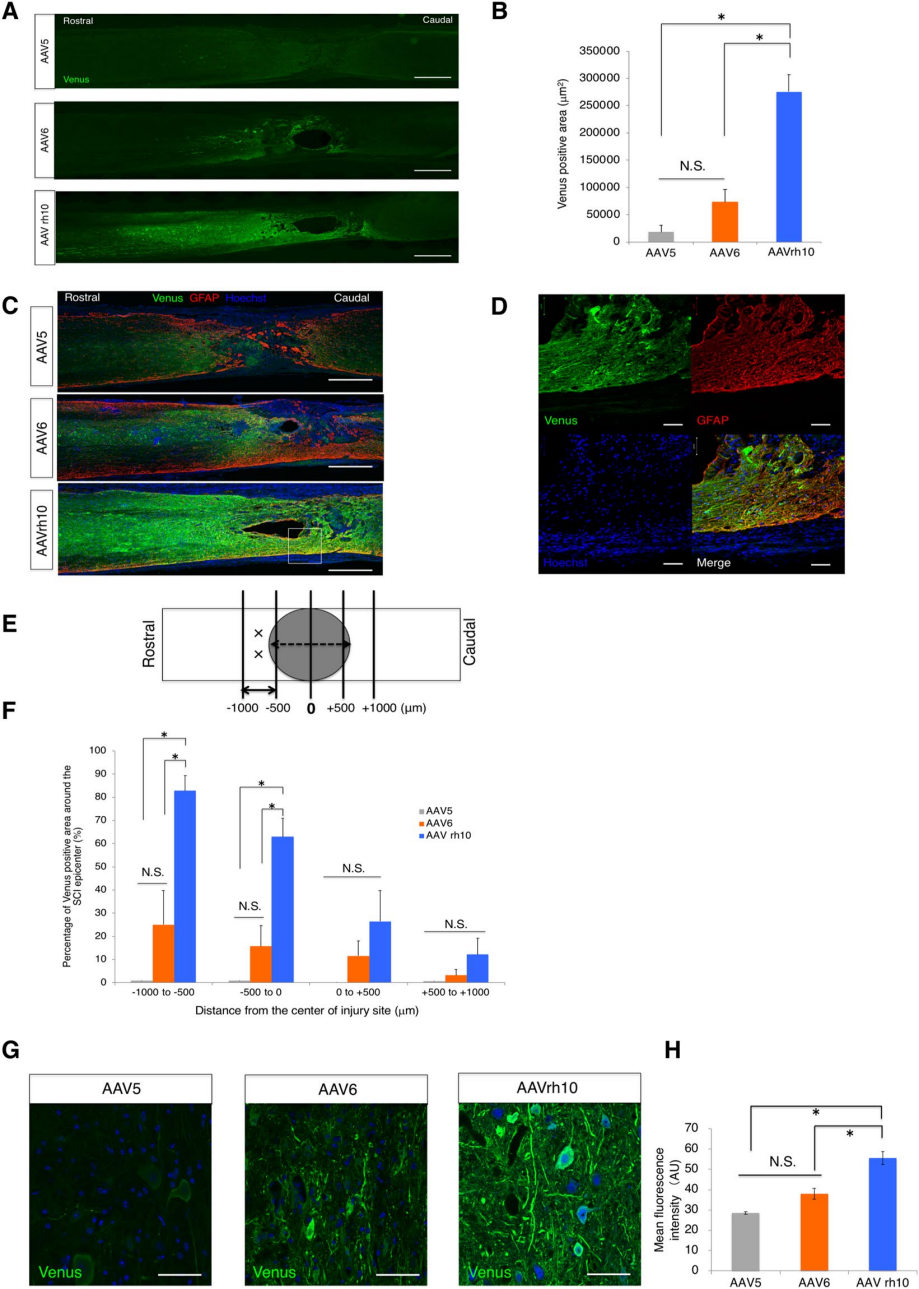
Characteristics of transgene expression in the injured spinal cord during the chronic phase. Sections were immunostained with an anti-GFP antibody. (**A**) Representative immunostained images of the spinal cord after 6 weeks of AAV-*ff*Luc injection. Scale bars, 500 μm. (**B**) Quantification of the transduced area of AAV-*ff*Luc in the chronically injured spinal cord. (**C**) Representative images of the differences in transduction around the injury epicentre, which was immunostained with GFAP to outline the lesion area. Note that the vector was injected only rostrally to the epicentre. Scale bars, 500 μm. (**D**) Higher magnification of the area in the square. Colocalization of Venus and GFAP was observed. Scale bars, 50 μm. (**E**) Schematic representation of the quantification, with a focus on transduction around the injured region. The grey area represents the diameter of the IH impactor tip (1250 μm). X points represent injection sites. Zero points indicate the centre of the diameter tip. We set the rostral side as negative and the caudal side as positive. Quantification was performed every 500 μm from the rostral and caudal sides. (**F**) Quantification of the percentage of Venus-positive area/spinal cord area in the 500 μm width around the SCI epicentre. (**G**) Representative images of *ff*Luc-driven fluorescence after the transduction of each AAV serotype. Scale bars, 50 μm. (**H**) Quantification of the fluorescence intensity of Venus immunostained with an anti-GFP antibody. p < 0.05 and not significant (N.S.) according to one-way ANOVA with the Tukey-Kramer test. Data are presented as the mean ± SEM.

In previous reports, AAV vectors were also characterized by measuring the reporter-positive area and the intensity of reporter activity after injection of AAV vectors^36,47^. Therefore, we examined the area expressing Venus and its fluorescence intensity to investigate the transduction of AAV vectors in chronic SCI. The area of the AAVrh10-driven reporter was significantly broader than that of the other vectors, as shown in Fig. 5A and B (AAV5: 18243 ± 12554 μm^2^, AAV6: 73266 ± 22392 μm^2^, AAVrh10: 275461 ± 32013 μm^2^). In addition, the Venus-positive area of AAVrh10 was conspicuously observed in the residual tissues around the epicentre, even on the opposite side of the injection point, as shown in Fig. 5C. Interestingly, we found colocalization of Venus with glial fibrillary acidic protein (GFAP) (Fig. 5D), suggesting that AAVrh10 transduced astrocytes around the SCI. For further analysis, area quantification was performed with a focus on the injury site. The size of the epicentre was estimated based on the diameter of the impactor tip (Fig. 5E), and the percentage of the Venus-positive area within the residual spinal cord tissues was calculated. Again, the expression area of AAVrh10 was significantly broader than that of the other serotypes in all sliced areas at −1000 to −500 μm from the injury epicentre (Fig. 5F). These results indicate that AAVrh10 could be effective for gene delivery in chronic SCI. With respect to fluorescence intensity, we measured the mean value of each captured image. Quantification analysis revealed that AAVrh10 showed a significantly higher fluorescence intensity than the other vectors, suggesting that AAVrh10 provides a high level of transgene expression.

## Discussion

In the present study, we injected various AAV vectors to identify the serotype most suitable for gene transfer in the injured spinal cord during the chronic phase. Prior to this study, the validity of the reporter system among the different serotypes was examined in the intact spinal cord. Although the transduction itself was successful, the level of transduction was different. The group size (n = 1) in this study was certainly a limiting factor. However, the transduction characteristics of each of the selected serotypes were broadly similar to those reported in previously published data^36–39^ and the results were cautiously examined. In the injured spinal cord during the chronic phase, we found that AAVrh10 was an appropriate serotype based on the evaluation using BLI and its broader cellular tropism for neurons, astrocytes and oligodendrocytes (Fig. 4B,D and F). In addition, transduction of AAVrh10 was achieved in a larger area than that of other serotypes (Fig. 5B). In particular, reporter expression of this serotype was prominent around the injury epicentre and in the surrounding area, and this feature was not observed for the other vectors (Fig. 5F). Furthermore, the fluorescence intensity was significantly higher with this vector (Fig. 5H). Thus, AAVrh10 injection is expected to yield strong genomic expression at the lesion site during the chronic phase and could therefore be a promising therapeutic tool for effectively delivering genes to overcome the unfavourable environment for spinal cord regeneration.

To analyse the transgene expression of different serotypes, we evaluated each serotype by quantification through immunohistochemistry^38,48^ and BLI, which have been shown to correlate with the protein expression of luciferase^49^. The dose-response relationship of photon counts indicated that BLI accurately measured the outcomes (Supplementary Fig. 3). To further understand AAV tropism, it is desirable to clarify the mechanism of serotype-dependent effects through the examination of other indexes, such as the vector genome copy number in target cells^45,49^.

Petrosyan *et al*. examined the availability of AAV in SCI during the hyper-acute phase. The authors reported the potential benefit of using AAVrh10 in terms of higher tropism not only for neurons but also for glial cells^38^. However, only semi-quantitative analysis was performed for the glial lineage, and the transduction efficiency for astrocytes was unclear. In this study, we demonstrated favourable tropism for various cell types and efficient gene transfer of AAVrh10 during the chronic phase. A treatment for chronic SCI will have more impact due to the limited therapeutic options, despite the larger number of patients in the clinical setting^50^. We hope to develop a new strategy to apply AAVrh10 for the management of this challenging pathological condition.

Other than serotypes, the tropism of AAV has been reported to be influenced by various factors including animal age, promoters, the route of administration, and viral dose^48,51–55^. Although we chose the ubiquitous CMV promoter to examine the tropism of AAV serotypes to spinal cord tissue based on previous reports^37,38,49^, selective gene transfer to target cells in the CNS has been achieved using cell type-specific promoters^54,55^. Therefore, in addition to the selection of optimal serotypes, the appropriate selection of promoters can further improve gene therapy using AAV and make it more broadly applicable in different fields. Additionally, promising newly engineered AAV variants designed for specific purposes, such as AAV2-retro^56^, AAV-TT^57^, and AAV-PHP.B^58^, all of which need to be evaluated to enhance the treatment potential of AAV vectors in the future.

AAV vectors targeting the CNS are mainly administered through intraparenchymal, intrathecal, or intravascular injection^36,47,49,59^. When AAVs are injected intravenously or intrathecally, gene transfer has been known to occur widely throughout the CNS. Although this is a preferable feature when genetic intervention is required in cells throughout the CNS, such as neurodegenerative disorders, unnecessary transgene expression in multiple non-targeted organs^49,59^ and hepatic dysfunction have been raised as possible concerns with excessive doses of AAV vectors^60^. Optimizing the dose can improve the safety of systemic administration^61–63^. Regarding direct intraparenchymal injection, although it is likely an invasive method for the spinal cord, there has been no report of obvious adverse events such as neurological deficits after the procedure^38^. In terms of safety, fewer viral particles are needed for intraparenchymal injection than for intravascular administration^49^. In addition, particularly in the chronic phase of SCI, there are chemical and physical obstacles to regeneration, such as scar formation and axonal inhibitory factors, which need to be overcome^40^. We believe that intraparenchymal injection allows us to administer the viral vector directly and at sufficient concentrations to ensure that therapeutic genes can be introduced more effectively into the injury site. Therefore, we selected intraparenchymal administration in this study considering its efficiency and safety.

To deliver therapeutic molecules to the target tissues, previous studies reported using gel foam or intrathecal catheter implantation to acquire beneficial effects following SCI^64,65^. However, these approaches required repetitive replacement surgeries for long-term administration because the sustained release period was limited^64^. These procedures were highly invasive and consequently increased the risk of infection^66^. Considering these adverse aspects, gene therapy would be an ideal option for the management of SCI since it has the potential to allow long-term expression of targeted genes with constitutive activation after a single administration. From our results, AAV may be a preferred platform for *in vivo* gene therapy among various viral vectors due to its safety and efficiency of transduction^67^.

For clinical application, quality control of viral vectors is an inevitable issue. In particular, purification is a critical step to generate better quality AAV vectors. The formation of empty capsids, which lack the vector genome, is one of the unique features of AAV vectors. It has been reported that empty capsids cause innate and adaptive immune responses^68,69^ to AAV and may inhibit transduction processes^70^. They can be distinguished from full capsids by electron microscopy^71^. Although the ratio of empty to full capsids did not seem to affect the transduction efficacy in the present study, this issue should be addressed prior to large-scale manufacturing for clinical use (Supplementary Fig. 2). Further development of purification methods and quality control are desirable.

Clinical trials using AAV have already been performed in several diseases^30–34,72,73^. This promising tool may also be applied for the treatment of human SCI. Although we demonstrated the availability of AAVrh10 in a mouse model of chronic SCI, the transduction profile and tropism of AAV could vary depending on the animal species^74^. For example, AAV5 displayed low-level transduction efficacy in the rodent brain^75^, while it showed high expression in the primate brain^76^. For future clinical application in humans, further investigation is necessary using larger mammals, in which SCI models have already been established^13^.

## Materials and Methods

### Cell culture

HEK293T cells (TaKaRa, Kusatsu, Japan) were cultured on dishes or plates in DMEM (Nacalai Tesque, Kyoto, Japan) supplemented with 10% FBS (Thermo Fisher Scientific), penicillin/streptomycin and L-glutamine. Transfections were performed using polyethylenimine (1 mg/mL, Polysciences, Warrington, PA, USA). For the acquisition of fluorescence images, the cells were observed with an EVOS fluorescence microscope (Thermo Fisher Scientific, Waltham, MA, USA) at 19 h after transfection. Images were analysed using ImageJ/Fiji software (NIH, Bethesda, MD, USA). For detection of luciferase activity, the cells were extracted with either RIPA buffer or ONE-Glo (Promega, Fitchburg, WI, USA) at 19 h post transfection. Bioluminescence was measured by using a GLOMAX luminometer (Promega).

### Plasmids

A Venus-luciferase 2 reporter gene cassette (ffLuc-cp156, gifted from Dr. Atsushi Miyawaki at RIKEN) was inserted into pAAV-MCS (Cell Biolabs, San Diego, CA, USA), which contains AAV2 ITRs, the CMV promoter, the human β-globin intron and the human growth hormone polyadenylation signal. Plasmids containing both Rep and Cap genes for AAV serotypes 1, 2, 3, 4, 5, and 6 were purchased from Cell Biolabs. To package the recombinant AAVs, including serotypes 7, 8, 9, and rh10, sequence information for each Cap gene was obtained from GenBank (NC_006260, NC_006261, AY530579 and AY243015, respectively) and was further synthesized (Eurofins Genomics, Tokyo, Japan). The synthesized DNA fragments were subsequently subcloned into the AAV Rep-Cap plasmid (Cell Biolabs).

### Generation of recombinant AAV vectors

HEK293T cells were transfected with three plasmids providing the AAV genome, Rep-Cap genes and adenovirus helper genes (pHelper, Cell Biolabs) by using polyethylenimine (1 mg/mL, Polysciences). Purification of AAV vectors was performed using an AAVpro purification kit (all serotypes) (Takara), including AAV extraction solution, precipitator solution, Millex-HV 0.45 μm, Amicon Ultra-15 (100 kDa), suspension buffer, and Cryonase™ Cold-active Nuclease, following the manufacturer’s instructions. Briefly, twenty-four hours after transfection, the culture medium was replaced with DMEM supplemented with 2% FBS. The cells were then harvested with 0.5 M EDTA and collected by centrifugation at 48 h after transfection. The cell pellets were extracted by AAV extraction solution (TaKaRa). For removal of genomic and plasmid DNA, the cell extracts were incubated with Cryonase™ Cold-active Nuclease (TaKaRa) for 1 h at 37 °C. Subsequently, contaminants were precipitated with precipitator solution (TaKaRa) and removed by centrifugation. The quality of the prepared vector was further examined by the purity and ratio of VPs (Supplementary Fig. 1). The supernatant was then passed through Millex-HV 0.45 µm filters (MilliporeSigma, Burlington, MA, USA). Finally, AAV preparations were concentrated using ultrafiltration units with a molecular weight cutoff of 100 kDa (MilliporeSigma) and reconstituted in suspension buffer (TaKaRa). Purified AAVs were stored at -80 °C before use. Following purification, titres were determined via quantitative PCR by using ViiA7 (Thermo Fisher Scientific). The primers were designed to specifically recognize the human growth hormone polyadenylation signal (forward, 5′-CCTGCGGGGTCTATTGGGAA-3′; reverse, 5′-GAGAATCGCTTGAACCCAGGA-3′).

### Phylogenetic analysis

The VP1 protein sequences encoded by 10 Cap genes (AAV1, 2, 3, 4, 5, 6, 7, 8, 9, and rh10) were aligned with the ClustalW algorithm (http://www.genome.jp/tools-bin/clustalw). A phylogenetic tree was then drawn using the calculated multiple alignment.

### Transmission electron microscopy (TEM) analysis

The negative staining sample preparation was performed according to the previous reports^71,77,78^. Briefly, the AAV was fixed with 2.5% glutaraldehyde (Electron Microscope Science) for 3 hours at 4 °C and was well mixed with vortex. Total 2 μL of fixed virus solution from each serotype group was applied on the copper mesh grids (VECO #400) covered with the carbon-coated formvar membrane and was incubated for approximately 1 min. Cupper grids were washed with diluted water at room temperature for 1 min and were stained with 1% filtered uranyl acetate. After removing excessive uranyl acetate with filter paper and the grids were left at room temperature to air dry completely for 30 min. The negatively stained virus samples were observed under a TEM with a 2K-by-2K charge coupled device [CCD] camera (JEOL, JEM-1400plus) at a x 60,000 magnification. 90 images were randomly acquired from the grids. The ratio of empty to total capsids and the percentage of empty capsids were quantified by counting the positively (or negatively) stained particles from micrographs.

### Mice

Female C57BL6 albino mice (8 weeks old, 18–20 g; Oriental Kobo, Tokyo, Japan) were used for injection into the intact spinal cord, and female C57BL6 mice (8 weeks old, 18–20 g; Oriental Kobo, Tokyo, Japan) were used for injection into the chronically injured spinal cord. The animals were housed in groups of no more than five per cage with a 12:12 light-dark cycle and ad libitum access to standard rodent chow and water in a temperature- and humidity-controlled environment. All animal experimental procedures were performed in accordance with the Guide for the Care and Use of Laboratory Animals (National Institutes of Health, Bethesda, MD, USA). All experiments were approved by the Keio University Institutional Animal Care and Use Committee in accordance with the Institutional Guidelines (approval number: 13020(3)).

### Surgery (SCI + AAV injection)

For experiments involving the intact spinal cord, each serotype of AAV-*ff*Luc (7.38 × 10^9^ vg/μL, 500 nL × 2 points, 100 nL/min) was injected intraparenchymally at the level of the 10th thoracic spinal cord through a glass pipette connected to a microinjector (BJ-110; BEX, Japan) (n = 1, each serotype). For the experiments involving the chronically injured spinal cord, contusion SCI was induced using an IH impactor (a force-defined impact (60 kdyn) with a stainless steel-tipped impactor; Precision Systems and Instrumentation, Lexington, KY, USA) at the level of the 10th thoracic spinal vertebra in the spinal cords of adult female C57BL6 mice. Six weeks after SCI, AAV vectors were injected on the immediate rostral side of the lesion epicentre in the same manner described above (n = 4 for each serotype).

### BLI

A Xenogen-IVIS spectrum-cooled, charge-coupled device (CCD) optical macroscopic imaging system (Caliper Life-Science, Hopkinton, MA, USA) was used for BLI. The mice were anaesthetized with 2% isoflurane and oxygen. Imaging was performed *in vivo* five minutes after i.p. injection of D-luciferin (0.3 mg/g body weight) with the field-of-view set at 13.2 cm, as the photon count was most stable during this period. The intensity peaked between 10 and 30 minutes. All images were measured with Living Image software, and the optical signal intensity was expressed as the photon count in units of photons/sec/cm^2^/str. Each result was displayed as a pseudo-coloured photon count image superimposed on a grey-scale anatomic image. A region of interest was defined in the area injected with AAV, and all values in the same region of interest were elucidated to quantify the light measured. Animals were imaged on a schedule of 1, 2, and 3 weeks after AAV injection to study the intact spinal cord and 3, 5, and 6 weeks after injection to study the chronically injured spinal cord.

### Tissue processing

Animals were anaesthetized and transcardially euthanized with 0.1 M PBS containing 4% PFA at 6 weeks after injection. The spinal cords were removed, post-fixed overnight in 4% PFA, and soaked overnight in 10% sucrose, followed by 30% sucrose. The samples were then embedded in Optimal Cutting Temperature (O.C.T.) compound (Sakura FineTechnical Co., Ltd., Tokyo, Japan), frozen, and sectioned in the sagittal plane at a 16 μm thickness on a cryostat (CM3050S, Leica Microsystems, Wetzlar, Germany).

### Immunohistochemistry

The tissue sections were stained with the following primary antibodies for immunohistochemistry: anti-GFAP (rat IgG, 1:200, Thermo Fisher Scientific #13-0300), anti-glutathione S-transferase-pi (GST-pi, rabbit IgG, 1:500, MBL, Nagoya, Japan, #312), anti-neuronal nuclei (NeuN, mouse IgG, 1:500, MilliporeSigma, MAB377C3), anti-green fluorescent protein (GFP, goat IgG, 1:1000, Rockland, Limerick, PA, USA, 600-101-215), anti-actin, α-smooth muscle-Cy3 (SMA, mouse IgG, 1:400, Sigma-Aldrich, St. Louis, MO, USA, C6198) and anti-CD31 (rat IgG, 1:50, BD Biosciences, San Jose, CA, USA, 550274).

Nuclei were stained with Hoechst 33258 (10 μg/ml, Sigma-Aldrich). The secondary antibodies were as follows: Alexa Fluor 488 donkey anti-goat IgG (1:1000, Thermo Fisher Scientific, A11055), which was used for the quantification analysis, Alexa Fluor 488 goat anti-rabbit IgG (1:1000, Thermo Fisher Scientific, A11034), Alexa Fluor 594 donkey anti-rabbit IgG (1:1000, Thermo Fisher Scientific, A21207), Alexa Fluor 555 donkey anti-rat IgG (1:1000, Abcam, Cambridge, UK, ab150154), and Alexa Fluor 647 goat anti-rat IgG (1:1000, Thermo Fisher Scientific, A21247). Samples were visualized on a fluorescence microscope (BZ 9000; Keyence Co., Osaka, Japan) or a confocal laser-scanning microscope (LSM 700, Carl Zeiss, Jena, Germany). In all sagittal spinal cord sections, the left side indicates the rostral side, and the upper side indicates the dorsal side.

### Quantification of images

#### Cellular tropism

More than five regions around the SCI expressing Venus were captured in each section at a 40x magnification with a confocal laser-scanning microscope (LSM 700). The number of cell-specific marker-positive cells, such as NeuN and GST-pi, was counted in each section. Of those marker-positive cells, the number of Venus-positive cells was counted and represented as a percentage. For colocalization with GFAP, the Venus-positive area that was also marker-positive was measured using ImageJ/Fiji in each section and represented as a percentage.

#### Area expressing Venus

All images that were used to measure the area expressing Venus were captured with a fluorescence microscope (BZ 9000), with the same parameters, including exposure time and magnifications, for each section. The transduction area per section for one complete set was measured using ImageJ/Fiji, as described previously^38^, with slight modifications. Briefly, the thresholded pixel area was calculated by eliminating the background and quantifying the Venus-positive area.

#### Transduction around the injury epicentre

All images that were used to analyse transduction around the injury epicentre were captured with a fluorescence microscope (BZ 9000). We defined the centre of the IH impactor tip (diameter 1250 μm) as the centre of the SCI and divided the area around the SCI into 4 columns with widths of 500 μm. Quantification was calculated for every column from the rostral to caudal side. The Venus-positive area was measured as described above and was represented as a percentage of the Venus-positive area/total area of residual spinal cord in the 500 μm width. The residual tissue was characterized using GFAP to outline the lesion area.

#### Fluorescence intensity

All images for evaluation were captured with a confocal laser-scanning microscope (LSM 700), with the same parameters, including exposure time and magnifications, for each section. The fluorescence intensity was amplified using an anti-GFP antibody^79^ and quantified as described previously^48^ with slight modifications. Briefly, more than 5 areas were randomly selected in the Venus-positive area, and the mean fluorescence intensity for each section was measured after eliminating background using ImageJ/Fiji.

### Statistical analysis

The statistical significance of differences between experimental conditions was determined by one-way ANOVA, followed by the Tukey-Kramer test. The level of significance was set at p < 0.05, and data are presented as the mean ± SEM.

## Supplementary information

Supplementary information
