# Supplementary material for: The adeno-associated virus rh10 vector is an effective gene transfer system for chronic spinal cord injury
Source: Sci Rep. 2019 Jul 8;9:9844. doi: 10.1038/s41598-019-46069-z (PMC6614469; doi:10.1038/s41598-019-46069-z)

Title

The adeno-associated virus rh10 vector is an effective gene transfer system for chronic spinal cord injury

Authors

Yutaka Hoshino[1,2], Kenji Nishide[2], Narihito Nagoshi[1], Shinsuke Shibata[2,3], Nobuko Moritoki[3], Kota Kojima[1], Osahiko Tsuji[1], Morio Matsumoto[1], Jun Kohyama[2*], Masaya Nakamura[1*], and Hideyuki Okano[2,3*]

Affiliation

[1]Department of Orthopedic Surgery, Keio University School of Medicine, 35 Shinanomachi, Shinjuku-ku, Tokyo, 160-8582, Japan

[2]Department of Physiology, Keio University School of Medicine, 35 Shinanomachi, Shinjuku-ku, Tokyo, 160-8582, Japan

[3]Electron microscope laboratory, Keio University School of Medicine, 35 Shinanomachi, Shinjuku-ku, Tokyo 160-8582, Japan

Correspondence

Jun Kohyama (jkohyama@a7.keio.jp), Masaya Nakamura (masa@a8.keio.jp), or Hideyuki Okano (hidokano@a2.keio.jp)

Supplementary Figure legends

Supplementary Figure 1-3

SUPPLEMENTARY FIGURE LEGENDS

Supplementary Figure 1. Purification of each AAV vector

To assess the purity of the viral vectors, each serotype was separated by SDS–PAGE followed by Coomassie Brilliant Blue staining. The three bands represent the capsid proteins VP1, VP2, and VP3 at an approximate ratio of 1:1:10. M lane, protein size marker.

Supplementary Figure 2. Electron micrographs of AAV particles

The ratio of empty to full AAV particles was examined using electron microscopy after negative staining with 1% uranyl acetate. Empty capsids (black arrowheads) were identified by an electron-dense circle at the centre of the capsid. White arrowheads indicate full capsids. The percentages of empty capsids were as follows: AAV5 = 20.9%, AAV6 = 70.0%, AAVrh10 = 60.3%. Scale bars, 100nm.

Supplementary Figure 3. Bioluminescence imaging following AAVrh10 injection into the spinal cord of chronic spinal cord injury model mice at varying doses.

Different doses of AAV-*ff*Luc serotype rh10 (7.38 $x10^{8}$ vg/μL, 7.38 $x10^{9}$ vg/μL, and 7.38 $x10^{10}$ vg/μL; n = 2, 3, and 2 animals, respectively; total injection volume = 1 μL per animal) were injected into the spinal cord of chronic spinal cord injury model mice. AAV injections and BLI were performed as described in the Materials and Methods section. **(A)** Representative *in vivo* BLI of a mouse 6 weeks after AAV-*ff*Luc serotype rh10 injection. **(B)** Photon counts correlated linearly with the viral vector dose of AAV rh10 at 6 weeks after injection.

Supplementary Figure.1

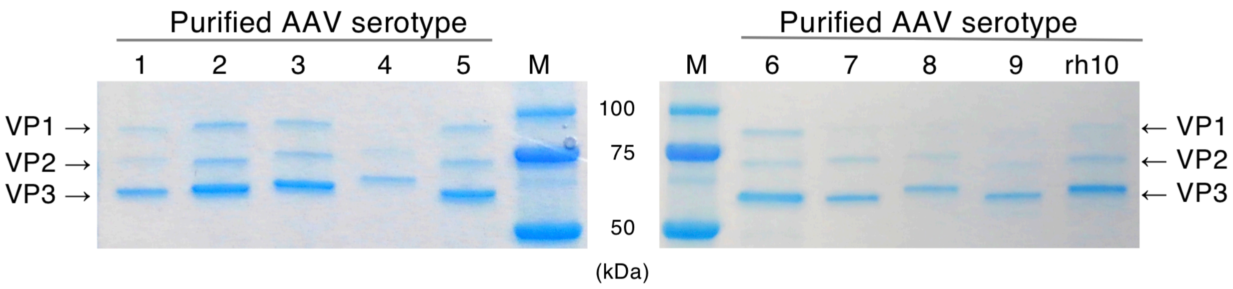

Supplementary Figure.2

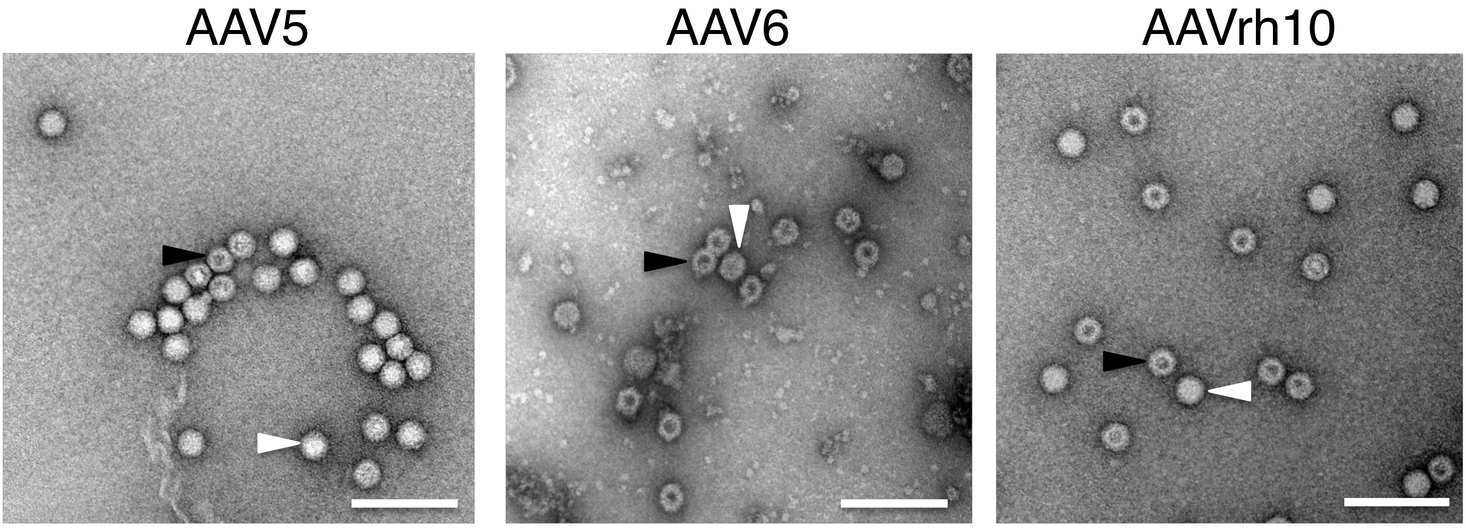

Supplementary Fig. 3

A

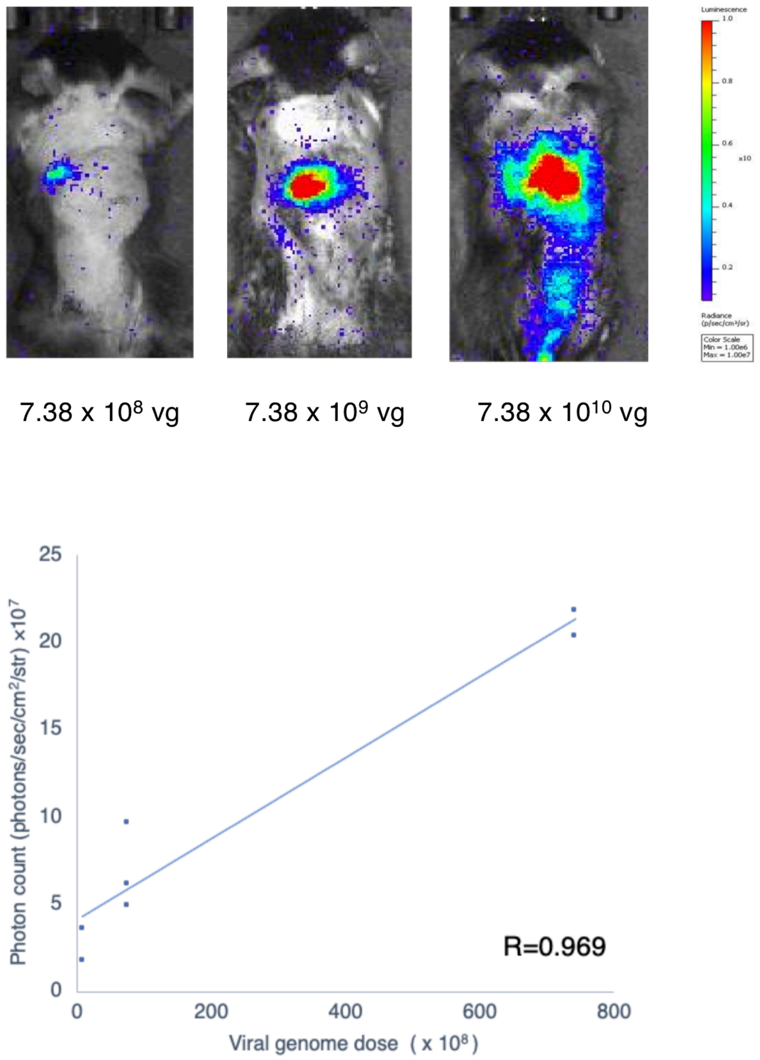

Supplement: Supplementary file 1 — Supplementary information [file 41598_2019_46069_MOESM1_ESM.pdf]
